# Psychological distress among undergraduate health sciences students in Uganda

**DOI:** 10.4102/phcfm.v17i1.4749

**Published:** 2025-03-26

**Authors:** Nakitende Naswiibah, Richard Muhindo

**Affiliations:** 1 Department of Nursing, College of Health Sciences, Makerere University, Kampala, Uganda

**Keywords:** psychological distress, health science, students, undergraduate, university

## Abstract

**Background:**

Psychological distress (PD) is a prevalent concern among undergraduate health science students globally. Despite this, data specific to Uganda is limited.

**Aim:**

This study assessed the prevalence of PD among undergraduate health sciences students in Uganda.

**Methods:**

We obtained data on the psychological distress burden using self-administered DASS-21 questionnaires. Data were analysed using SPSS version 20.

**Results:**

We enrolled 398 participants, of whom 217 (54.5%) were males. The median age of the participants was 22 years (interquartile range [IQR], 21 to 24). Of the participants, more than half (57%) had moderate to severe symptoms of anxiety. Nearly half of them (42%) reported moderate to severe symptoms of depression, while 26% of the students had moderate to severe symptoms of stress.

**Lessons Learnt:**

This study highlights significant psychological distress among health science students at Makerere University, with high levels of anxiety, depression and stress. It emphasises the need for improved mental health support in academic settings, aligning with the *African Journal of Primary Health Care & Family Medicine*’s focus on contextual healthcare challenges.

## Introduction

### Social value

Globally, psychological distress (PD) has been a longstanding issue affecting university students.^1,2^ As a major aspect of mental health, it encompasses a range of emotional suffering characterised by symptoms of depression (e.g., loss of interest, unhappiness, desperateness) and anxiety (e.g., restlessness, feeling tense).^3^ Among students, poor academic outcomes and problematic health behaviours, for example, illicit drug use and risky sexual practices are linked to distress.^4,5^ Additionally, over time, PD is associated with mental health disorders, such as attention deficit disorder, anxiety disorders and depression as well as physical health problems like heart diseases.^6,7^

### Scientific value

Psychological distress is an indicator of students’ psychological well-being.^8^ However, systematic reviews and meta-analyses have found high PD prevalence, 51% (CI 95% [44.0–58.0]), positively associated with gender among health sciences students.^9^ Similar findings (prevalence ranges of 15.7% to 98%) were reported from individual studies conducted in China, Malaysia and Nigeria.^5,10^ In Uganda, data on PD among health sciences students are limited. While high PD prevalence was reported among secondary school students, the context may differ in Ugandan universities.

Additionally, the rigour of education curricula could differ across disciplines. Health sciences disciplines are known for their rigorous academic and clinical demands, requiring students to balance extensive coursework with practical rotations. Health sciences students are a future key resource for the health care system, and understanding the burden of PD among this cohort has wider implications to highlight the need for appropriate policies and support services.

## Aim

This study aimed to document the burden of PD among undergraduate health sciences students in Uganda.

## Research methods and design

### Study design

This study employed a cross-sectional design.

### Setting

The study was conducted at Makerere University College of Health Sciences (MakCHS),^11^ located on Mulago Hill, Mulago, 3 km from Kampala city, Uganda.^11^ Makerere University College of Health Sciences, the largest and most prestigious health training institution in Uganda, is renowned for its academic excellence and rigorous standards. It is one of 10 colleges at Makerere University. The rigorous coursework, rotations and exams at MakCHS create significant academic pressure. Combined with its prestigious reputation, this environment amplifies PD among students, emphasising the need for mental health support services.

### Study population and sampling strategy

To estimate the study sample size, we aimed for 5% precision, assuming a PD prevalence of 40.2% from a previous study among students in Uganda.^12^ Using the Cochran formula, a total of 398 students were estimated (two-sided test at 95% significance, 5% margin of error). Makerere University College of Health Sciences had 1710 undergraduate students across 12 programmes: MBChB (750, 43.9%), BPHA (200, 11.7%), BDS (125, 7.3%), BEHS (120, 7.0%), BSB (120, 7.0%), BNUR (80, 4.7%), BMR (80, 4.7%), BBI (80, 4.7%), BCYT (60, 3.5%), BSLT (40, 2.3%), BOPT (40, 2.3%) and BDLT (15, 0.9%). Stratified sampling with proportion-to-size allocation was used to ensure programme and academic year representation. Class representatives, serving as research assistants, facilitated recruitment, ensuring inclusivity and minimising bias while adhering to ethical standards. This comprehensive approach enabled a representative sample of the student population.

### Data collection

The Depression Anxiety Stress Scale (DASS-21), a 21-item self-report tool designed to measure symptoms of depression, anxiety and stress, was used to obtain data on symptoms of depression, anxiety and stress. It uses a four-point (0–3) scale to assess the extent of symptoms experienced over the previous week and has been widely used among patients, occupational workers and students in a number of settings, including Africa.^13,14,15^. Studies in Uganda and Nigeria have confirmed its validity and reliability among medical students.^16,17,18^ Additionally, we obtained data on demographic characteristics of the respondents. The self-administered tool was distributed by trained course representatives.

### Data analysis

Data were analysed using SPSS version 20. Cronbach’s alpha assessed the reliability of the DASS-21 questionnaire, which has three subscales (depression, anxiety and stress), each containing seven items. Sum scores for each subscale were calculated, doubled and categorised into levels: (1) *depression*: normal (0–9), mild (10–13), moderate (14–20), severe (21–27), very severe (≥ 28); (2) *anxiety*: normal (0–7), mild (8–9), moderate (10–14), severe (15–19), very severe (≥ 20) and (3) *stress*: normal (0–14), mild (15–18), moderate (19–25), severe (26–33), very severe (≥ 34).^19^ Frequency distributions and percentages described socio-demographic characteristics and PD among students.

### Ethical considerations

Ethical clearance to conduct this study was obtained from the Makerere University School of Health Sciences Research Ethics Committee (No. MAKSHSREC-2023-500). Written consent was obtained from participants, and confidentiality was maintained by anonymising data and storing it on password-protected devices. All procedures adhered to the ethical standards of the Declaration of Helsinki (2013 revision).

## Results

### Population characteristics

A total of 398 students participated in the survey. The median age was 22 years (interquartile range [IQR], 21 to 24). Just over half (54.5%) were males, nearly half (44%) were Medicine (MBChB) students and 16.1% were orphans. Of the participants, 316 (79.4%) perceived the academic schedule as either difficult or somewhat difficult, and 129 (32.4%) described obtaining tuition as either difficult or somewhat difficult. Half (50%) of the participants reported difficulty accessing meals. Additionally, 19.6% reported current alcohol or drug use, 8.5% had a history of chronic illness, and 32% engaged in a betting practice. Less than half (32%) participated in regular physical activity (Table 1).

**TABLE 1:** Socio-demographic characteristics of the participants (*N* = 398).

Variables	*n*	%
**Gender**		
Female	181	45.5
Male	217	54.5
**Chronic illness**		
Yes	34	8.5
No	364	91.5
**Tuition accessibility**		
Difficult	56	14.1
Somewhat difficult	73	18.3
Neutral	123	30.9
Somewhat easy	60	15.1
Easy	86	21.6
**Academic schedule perception**		
Difficult	171	43.0
Somewhat difficult	145	36.4
Neutral	71	17.8
Somewhat easy	9	2.3
Easy	2	0.5
**Parental status**		
Alive	334	83.9
single orphaned	55	13.8
double orphaned	9	2.3
**Meals accessibility**		
Difficult	74	18.6
Somewhat difficult	125	31.4
Neutral	72	18.1
Somewhat easy	92	23.1
Easy	35	8.8
**Physical activity**		
Never	67	16.8
Seldom	114	28.6
Sometimes	120	30.2
Often	59	14.8
Always	38	9.5
**Alcohol or Drug use**		
Yes	78	19.6
No	320	80.4
**Betting practice**		
Never	271	68.3
Ever	126	31.7

Note: Age (years): Median = 22; IQR = 21–24.

IQR, interquartile range.

### Construct reliability

The internal consistency of the DASS subscales was evaluated using Cronbach’s alpha, with each subscale showing strong reliability (α > 0.71) (Table 2). Correlation analysis indicated a statistically significant relationship between the depression, stress and anxiety subscales.

**TABLE 2:** Correlation between the Depression Anxiety Stress Scale (DASS21) subscales.

DASS subscale	Cronbach’s alpha	Depression	Anxiety	Stress
Depression	0.80	1.00	-	-
Anxiety	0.71	0.60	1.00	-
Stress	0.75	0.62	0.50	1.00

### Symptoms of psychological distress (depression, anxiety and stress) among undergraduate health sciences students

A significant proportion of undergraduate health sciences students experienced moderate to severe symptoms of poor mental health, with 57% reporting anxiety, 42% depression and 26% stress, according to DASS-21 scores (Table 3). However, no statistically significant relationships were found between poor mental health and demographic characteristics of respondents (Table 4).

**TABLE 3:** Symptoms of psychological distress (depression, anxiety and stress) among university undergraduate health sciences students.

DASS-21 Scores	*n*	%
**DASS21, mean**		
Depression	12.7	-
Anxiety	13.9	-
Stress	11.3	-
Total_DASS score	37.9	-
**DASS-21 _depression**		
Normal (0–9)	150	37.7
Mild (10–13)	79	19.8
Moderate (14–20)	114	28.6
Severe (21–27)	25	6.3
Extremely severe (≥ 28)	30	7.5
**DASS-21_Anxiety**		
Normal (0–7)	127	36.1
Mild (8–9)	44	11.1
Moderate (10–14)	116	29.3
Severe (15–19)	45	11.4
Extremely severe (≥ 20)	64	16.2
**DASS-21_Stress**		
Normal (0–14)	231	58.6
Mild (15–18)	61	15.5
Moderate (19–25)	60	15.2
Severe (26–33)	35	8.9
Extremely severe (≥ 34)	7	1.8

DASS, Depression anxiety stress scale.

**TABLE 4:** Factors associated with symptoms of psychological distress (depression, stress and anxiety) among university undergraduate health sciences students.

Variables	Depression			Anxiety			Stress		
	aPR	95% CI	*p*	aPR	95% CI	*p*	aPR	95% CI	*p*
**Age (years)**									
18–27	0.959	0.601–1.533	0.862	1.018	0.650–1.594	0.937	0.983	0.595–1.625	0.946
28–36	-	Reference	-	-	Reference	-	-	Reference	-
**Gender**									
Female	0.990	0.831–1.179	0.909	1.014	0.858-1.198	0.871	1.048	0.869–1.263	0.622
Male	-	Reference	-	-	Reference	-	-	Reference	-
**Course offered**									
MBChB	1.004	0.668–1.511	0.983	0.918	0.635–1.329	0.651	0.873	0.572–1.331	0.527
BNUR	0.965	0.562–1.658	0.897	0.964	0.584–1.592	0.887	0.895	0.510–1.572	0.700
BPHA	0.955	0.601–1.518	0.847	0.840	0.548–1.289	0.425	0.947	0.587–1.525	0.822
BOPT	0.981	0.482–1.998	0.953	0.801	0.402–1.597	0.529	0.998	0.487–2.045	0.996
BMR	0.987	0.574–1.695	0.961	0.924	0.561–1.522	0.758	0.844	0.478–1.491	0.560
BSLT	1.156	0.579–2.308	0.682	0.706	0.339–1.467	0.350	1.156	0.574–2.331	0.684
BSB	1.028	0.621–1.702	0.916	0.911	0.572–1.451	0.695	1.065	0.634–1.788	0.813
BDS	0.969	0.591–1.587	0.899	0.890	0.565–1.402	0.615	0.967	0.583–1.603	0.896
BDLT	0.926	0.312–2.750	0.890	0.969	0.363–2.586	0.950	0.911	0.301–2.757	0.870
BEHS	0.875	0.521–1.472	0.616	0.875	0.546–1.403	0.579	0.803	0.464–1.392	0.435
BCYT	1.012	0.559–1.833	0.969	0.839	0.476–1.480	0.545	0.861	0.449–1.652	0.653
BBI	-	Reference	-	-	Reference	-	-	Reference	-
**Year of study**									
One	1.089	0.767–1.546	0.634	1.005	0.720–1.402	0.977	1.055	0.726–1.534	0.779
Two	1.120	0.790-1.588	0.524	1.054	0.758–1.467	0.753	1.058	0.729–1.534	0.768
Three	1.185	0.841–1.671	0.331	1.075	0.776–1.489	0.663	1.058	0.733–1.527	0.764
Four	1.045	0.731–1.494	0.807	1.013	0.722–1.419	0.942	1.063	0.728–1.553	0.752
Five	-	Reference	-	-	Reference	-	-	Reference	-
**Chronic illness**									
Yes	1.105	0.822–1.486	0.509	1.022	0.762–1.369	0.885	1.156	0.845–1.582	0.364
No	-	Reference	-	-	Reference	-	-	Reference	-
**Tuition accessibility**									
Difficult	0.939	0.689–1.281	0.691	1.034	0.771–1.386	0.824	0.888	0.642–1.229	0.474
Somewhat difficult	0.996	0.754–1.317	0.978	1.012	0.773–1.326	0.929	0.905	0.673–1.217	0.509
Neutral	1.007	0.788–1.286	0.958	1.023	0.807–1.296	0.852	0.880	0.678–1.143	0.337
Somewhat easy	0.995	0.742–1.334	0.973	1.003	0.755–1.331	0.985	0.943	0.689–1.290	0.713
Easy	-	Reference	-	-	Reference	-	-	Reference	-
**Academic schedule**									
Difficult	0.715	0.252–2.034	0.530	0.818	0.288–2.319	0.705	0.976	0.296–3.224	0.969
Somewhat difficult	0.706	0.249–2.000	0.512	0.805	0.285-2.274	0.682	0.932	0.283–3.069	0.907
Neutral	0.661	0.231–1.895	0.441	0.773	0.271–2.205	0.631	0.907	0.272–3.022	0.873
Somewhat easy	0.740	0.226–2.418	0.618	0.778	0.239–2.529	0.676	1.061	0.278–4.048	0.931
Easy	-	Reference	-	-	Reference	-	-	Reference	-
**Parental status**									
Alive	1.046	0.573–1.909	0.884	1.365	0.733–2.542	0.326	0.993	0.529–1.862	0.982
single orphaned	1.015	0.537–1.918	0.964	1.177	0.610–2.268	0.627	0.960	0.493–1.870	0.904
double orphaned	-	Reference	-	-	Reference	-	-	Reference	-
**Meals accessibility**									
Difficult	0.974	0.671–1.413	0.888	1.017	0.712–1.454	0.925	1.056	0.707–1.576	0.791
Somewhat difficult	0.918	0.645–1.306	0.634	0.995	0.712–1.392	0.979	1.021	0.700–1.489	0.913
Neutral	0.945	0.649–1.377	0.769	0.925	0.644–1.329	0.674	0.990	0.661–1.482	0.961
Somewhat easy	0.987	0.693–1.406	0.943	0.935	0.665–1.315	0.699	0.995	0.679–1.459	0.982
Easy	-	Reference	-	-	Reference	-	-	Reference	-
**Physical activity**									
Never	1.219	0.841–1.767	0.297	1.106	0.780–1.567	0.571	1.202	0.814–1.774	0.356
Seldom	1.174	0.828–1.664	0.368	1.040	0.750–1.442	0.814	1.056	0.730–1.528	0.773
Sometimes	1.128	0.792–1.607	0.505	1.017	0.731–1.416	0.919	1.151	0.794–1.669	0.457
Often	1.195	0.824–1.733	0.347	1.042	0.735–1.477	0.818	1.178	0.797–1.741	0.410
Always	-	Reference	-	-	Reference	-	-	Reference	-
**Alcohol/drug use**									
Yes	1.078	0.855–1.359	0.526	1.081	0.868–1.347	0.487	1.156	0.905–1.478	0.246
No	-	Reference	-	-	Reference	-	-	Reference	-
**Betting practice**									
Never	1.038	0.850–1.267	0.717	1.022	0.846–1.235	0.820	1.024	0.828–1.266	0.829
Ever	-	Reference	-	-	Reference	-	-	Reference	-

BCYT, cytotechnology; BBI, biomedical engineering; BSB, biomedical sciences; BEHS, environmental health science; BOPT, optometry; BPHA, pharmacy; BDLT, dental laboratory technology; BDS, dental surgery; BSLT, speech and language therapy; BMR, medical radiography; MBChB, medicine; BNUR, Nursing; Cl, confidence interval; aPR, adjusted prevalence ratio.

## Discussion

In this study, a high prevalence of PD was observed among undergraduate health sciences students, likely attributed to the demanding academic workload, high expectations and challenging coursework. These findings highlight the need for institutions to integrate mental health support into their curricula to help students manage stress effectively.

The prevalence rates in this study align with prior research in Uganda. Nantaayi et al.^12^ reported a prevalence of 40.2%, and Anyanwu^20^ found 57% prevalence during the pandemic.^12,20^ Pre-pandemic studies noted depression and stress prevalence rates of 21.4% and 57.4%, respectively, demonstrating the persistence of mental health challenges among students.^21,22^ Globally, PD has been linked to factors such as gender, academic stress, low physical activity, financial distress and substance abuse.^2,4,5,10,16,17^ However, these associations were not statistically significant in this study, possibly because of differences in populations or cultural contexts, highlighting the need for tailored interventions.

Our findings emphasise the need for stakeholders to address poor mental health by implementing targeted strategies: universities should establish mental health services like counselling and stress management workshops; policymakers should invest in services addressing academic stress and post-pandemic challenges while evaluating intervention effectiveness; researchers should conduct longitudinal studies to track mental health trends and assess interventions and students and the community should promote awareness campaigns to encourage help-seeking and normalise mental health discussions.

Our study utilised the standard, validated DASS-21 questionnaire and is the first, to our knowledge, to assess PD among undergraduate health science students in Uganda post-coronavirus disease 2019 (COVID-19). However, the study has limitations. The cross-sectional design prevents the evaluation of changes in PD over time, underscoring the need for future longitudinal studies to assess mental health trends and the long-term impact of university stressors. Additionally, recall bias from self-administered questionnaires was mitigated by pre-testing the tool, but future research should consider incorporating diverse data collection methods (e.g., interviews or focus groups) for deeper insights. Despite these limitations, our findings align with previous studies among undergraduate health science students.

## Conclusion

A high prevalence of PD was observed among undergraduate health science students. This highlights the need for strategies to address mental health, such as improving access to counselling, offering stress management resources and fostering supportive academic environments.
